# Estimating dewatering in an underground mine by using a 3D finite element model

**DOI:** 10.1371/journal.pone.0239682

**Published:** 2020-10-29

**Authors:** Litang Hu, Menglin Zhang, Zhengqiu Yang, Yong Fan, Jixiu Li, Hongliang Wang, Celestin Lubale

**Affiliations:** 1 College of Water Sciences, Beijing Normal University, Beijing, P.R. China; 2 Engineering Research Center of Groundwater Pollution Control and Remediation of Ministry of Education, Beijing Normal University, Beijing, P.R. China; 3 China ENFI Engineering Corporation, Beijing, P.R. China; 4 Jinchuan Group Co., Ltd, Beijing Road, Jinchuan District, Jinchang City, Gansu Province, P.R. China; 5 North China Engineering Investigation Institute Co., Ltd., Hebei Province, Shijiazhuang, P.R. China; 6 Technological Innovation Center for Mine Groundwater Safety of Hebei Province, Shijiazhuang, P.R. China; 7 Ruashi Mining SAS, Ruashi Mine Site, Lubumbashi, Lualaba, The Democratic Republic of Congo; China University of Mining and Technology, CHINA

## Abstract

Groundwater inflow to an underground mine will seriously affect its mining plan and engineering geology safety. Groundwater models are powerful tools commonly used in the mines to develop dewatering strategies. Many mines in the Kolwezi area have been present since the 1950s, and groundwater flow patterns have been significantly influenced by mining activities. A mining plan is developed for an underground mine with overturned syncline strata in Kolwezi, Congo. Previous groundwater models using layered homogeneous media lowered model accuracies. A new three-dimensional groundwater model using FEFLOW, consisting of a combined regionally and locally geology models integrating 16 hydrogeological cross-sections and borehole logging data, are formulated to predict the underground dewatering in the study area. A 31-days pumping tests with 3 pumping wells and 28 observation wells are carried out to estimate the hydrogeological properties. The simulated water level data match the observed data rather well. Under 8 scenarios of possible well designs, the model predicts a possible dewatering capacity greater 23,900 m^3^/d at the initial stage of mining. The concept of the model and its application can be a reference for other mines with complex geology for mining safety in the region of interest.

## Introduction

Mineral exploitation and mining activities play a vital role in the economic development of the Democratic Republic of Congo (DRC). According to the USGS 2015 Minerals Yearbook, the DRC accounted for 51% of Africa’s copper mine production, and the mining and mineral processing sector in the DRC accounted for an estimated 22.1% of its gross domestic product (GDP). Many of the most important mining operations in the DRC consist of copper and cobalt production in the copper-belt region of the southern Katanga province. The belt stretches for 250 km between Kolwezi and Lubumbashi. Industrial copper production started in 1911 with the Union Minière du Haut Katanga (which became Gécamines), and the Musonoi mine has been known for many years in the western part of the Katanga Copperbelt.

During mineral deposit exploitation, dewatering of subsurface strata is one of the most important tasks to ensure an engineering geologically safe and efficient mining operation [1]. Under complex geological condition, analytical solution [2, 3] and analytical element models cannot be available, and thus numerical models are efficient tools to provide a dewatering plan in porous-medium areas [4–7] and fractured and karst regions [8–11]. The zonation of hydrogeology parameters is usually made based on borehole data and data interpolation [12]. Unlike layered soils [13], zonation is hard to achieve by data interpolation for complex geological setting, such as overturned synclines. 3D geology models, which integrate many kinds of field investigation data, are necessary and usually used tools to demonstrate the distribution of formations in mines activities [14, 15]. Importantly, geological heterogeneity is associated with open mine voids, tectonic faults and fractures, and traditionally structured grids have great difficulty representing high heterogeneity. Geology models, from existing geological modeling software such as Petrel, GeoModeller and Leapfrog, provide a visual demonstration and knowledge of the distribution of formations, especially controlling aquifers or faults. However, few researches are concerned with combining groundwater flow models with 3D geology models in complicated aquifers. MODFLOW-USG [16] and FEFLOW 7 [17] have been developed to describe complex geology conditions with unstructured grid systems. Meanwhile, uncertainties are still present in aquifer geometry and its characteristics due to limited investigations and complexity of geological conditions. Hassen and Gibson *et al*. [18] construct a 3D geology model using GeoModeller software and evaluate the influence of aquifer connectivity and the role of faults and geology in groundwater flow within the aquifer system; however, to our knowledge, few groundwater flow models with coupled geology model are reported. The coupling of flow model and geology model are effective to improve the accuracy of numerical model because it provides both the visual display of geology conditions and groundwater flow pattern, and thus accurate estimation of dewatering will minimize the risks during the deep excavation of mining.

The Kolwezi megabreccia contains Cu-Co deposits hosted in folded and brittle-fractured structures of the Mines Subgroup. As indicated by the surface map and cross-sections [19–21], these structures are aligned more or less parallel to the E-W/60—SW-NE orientation of the thrust nappe [22, 23]. The Musonoi mine is a newly planned underground mine in the Kolwezi Copper Deposit, DRC. The syncline strata in the Musonoi mine are overturned, with complex geologic and hydrogeological conditions. Several mines are present in the Kolwezi nappe area, including the KOV (Kamoto, Oliveira and Virgule) open pit [24], the Kamoto underground mine, Kamoto East, the old Musonoi open pit, the Dikuluwe open pit, the Mashamaba West and East open pits, the Sicomines mine, and the Kolwezi copper mine. Intense mining activities alter the natural path of groundwater flow and strengthen the groundwater connections among different mines in the Kolwezi copper mine. Intense mining activities alter the natural path of groundwater flow and strengthen the groundwater connections among different mines in the Kolwezi nappe area. Groundwater flow models have been developed to explore the dewatering strategies in the Kolwezi nappe area. The first public computer modeling of groundwater flow [25] is performed for the Dikuluwe and Mashamaba West pits and indicates that the progress of dewatering in 1985 would not be satisfactory for the existing mining plans. Africa Geo-Environmental Services (Pty) Ltd. develops groundwater flow models that consider the dewatering of KOV, Kamoto East, Kamoto Underground, Dikuluwe, Mashamaba East and Mashamaba West in 2006. SLR Consulting (South Africa) (Pty) Ltd. takes the Kolwezi nappe area as the model area to estimate the dewatering in the newly planned Musonoi mine in 2013 and 2017 using FEFLOW software. Such models simplify the strata into layered properties, and the simplifications affect model accuracies to some extent.

Although a lot of field investigations are carried out in the local mine area, few are present at the regional scale. However, the model boundary is required to extend beyond the mine area so as to avoid the influences of adjacent mining activities and man-made boundary on the accuracy of groundwater flow model. So the main objectives of this research are to combine groundwater flow model with regional and local geology model, and then explore the dewatering in underground mine using the established model. First, a hydrogeological conceptual model is designed for the underground mining activities. Second, the 3D geology model is constructed at regional and local scales and thus the parameterization will be carried out. Then, data will be prepared and then the groundwater flow model will be calibrated. Last, the established model is used to predict the dewatering in the Musonoi mine. The results of this paper can be effectively used for references in developing an efficient dewatering program.

## Material and methods

### Study area

**Fig 1:**
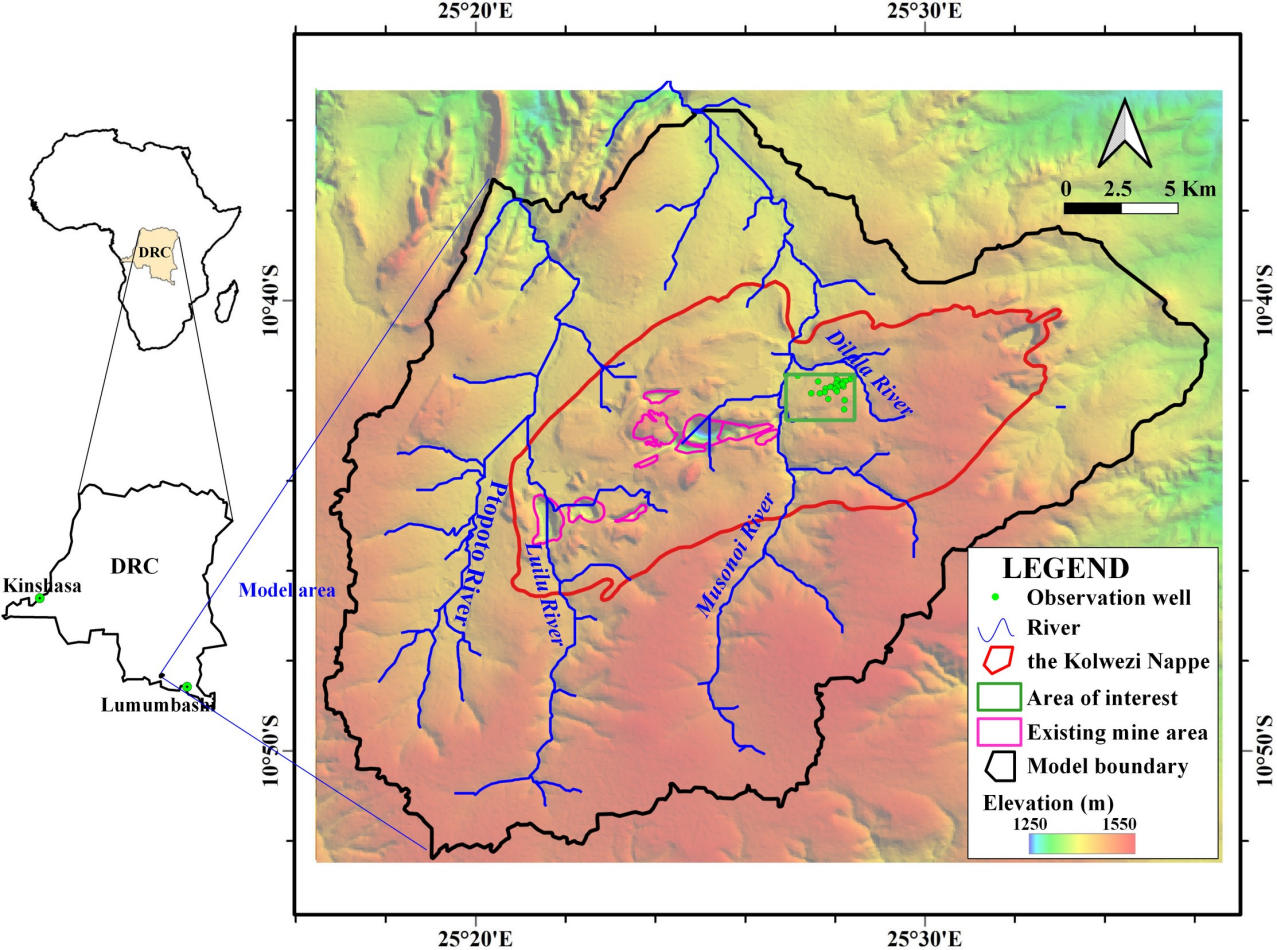
Location of the study area. Image created by the authors in QGIS 3.10 and Visio 2016; no copyrighted material was used.

The study area is located at the south of the equator in the Katanga plateau of the DRC (Fig 1). The study area has a savanna climate. The annual mean temperature is approximately 21.2°C, the highest temperature is approximately 31.9°C, and the lowest temperature is approximately 9.4°C. The average annual precipitation from 1979 to 2017 is approximately 1144.90 mm and the average annual evaporation is approximately 1860.00 mm. The maximum annual precipitation is 1826.00 mm (in 1986), and the minimum annual precipitation is 803.50 mm (in 1993). Precipitation mainly happens from November to March of the following year and accounts for more than 85% of the annual precipitation. The dry season is from May to September, with little monthly precipitation of less than 5 mm. The overall terrain is high in the south and low in the north, with varying elevations from 1250 m to 1550 m. There are three main rivers in the study area, the Luilu River, the Musonoi River and the Dilala River. The Luilu River (including the Ptopoto River) and the Musonoi River flow towards the north. Catchment areas for the Luilu and Musonoi Rivers are 236 km^2^ and 190 km^2^, respectively. The Dilala River surrounds the east and north sides of the mine area and finally joins the Musonoi River in the northwest of the mine area. During the dry season, the measured runoff of the Dilala River on the east side of the mine area is about 0.085 m^3^/s.

The strata in the study area are of the Late Proterozoic Katanga supergroup, which can be subdivided into the upper Kundelungu group and the lower Roan group (host strata). Regional geology is mainly controlled by the Katanga Fold—Nappe structure. The secondary structure in the nappe body is complex with folded fractures. The nappe structure has an important influence on the water content and spatial distribution of the aquifer. The strata in this area are mainly the Katanga series and the quaternary. The Katanga series mainly includes the Roan group (R), the Nguba group (Ng) and the Kundelungu group (Ku). The series of geology from the young to the old can be seen in Table 1.

**Table 1:** Regional stratigraphic list in the study area.

Series (From young to old)	Formation		Local Name	Brief Description	Approximated Thickness (m)
**Kundelungu**			Ku	Sediments	3000–5000
**Nguba**			Ng	Sandstone,shale	200–500
**Upper Roan**	R4		Mwashya	shale,siltstone, sandstone, dolomintes	50–100
	R3-2		Dipeta	Sandy shales	about 1000
	R3-1		RGS	Grey shales	100–200
**Lower Roan**	R2-3	Mines Group	CMN	Black calcareous siltstone	130
	R2-2		SDS	Dolomitic shales, black ore mineral zone (BOMZ)	50–80
	R2-1		SDB	Dolomitic shales, black ore mineral zone (BOMZ)	10–15
			RSC	Siliceous, vuggy dolomite	12–25
			RSF	Bedded dolomitic siltstone	5
			DSTRAT	Grey talcose sandstone	3
			RAT	Grey talcose sandstone	2–5
	R1		RAT2	Talcose sandstone	190
			RAT1	Talcose sandstone	40

### Conceptual model

Due to the long-term mining activities, the natural groundwater flow paths in each mine area are significantly altered. The hydraulic connections of the groundwater system in each mine area are much closer to the mining process due to the intense mining. Therefore, it is unreasonable to establish a numerical model of the groundwater flow in a certain mine area, and, based on the study of the digital elevation model of the study area, hydrology toolboxes in QGIS software are used to divide the watershed boundary. The model boundary, shown in Fig 1, considers the structure of the nappe body to minimize the influence caused by the setting of artificial boundaries. The west and south boundaries are set as natural watershed divides and are represented as no-flow boundaries. The northern and eastern boundaries of the model area are set as flow boundaries and are represented in the model as the Neumann boundaries. As drilling boreholes indicate, the depth of fracture and tectonic development is extended to 1000 m below the surface. The bottom of the model is set at the depth of 2000 m below the surface, and the bottom can thus be represented as a no-flow boundary. To take the complexity of the stratum change into account, the study area is divided into 18 layers of hydrogeological structure. The total thickness of the studied layer is 2000 m, and the thicknesses of the divided layers are, in order from the ground surface, 50 m, 50 m, 50 m, 50 m, 50 m, 50 m, 50 m, 50 m, 50 m, 50 m, 100 m, 100 m, 100 m, 100 m, 100 m, 200 m, 300 m and 500 m. The upper boundary mainly receives the infiltration from precipitation, and groundwater outflows to the mines.

## Model development

### Flowchart of the study

**Fig 2:**
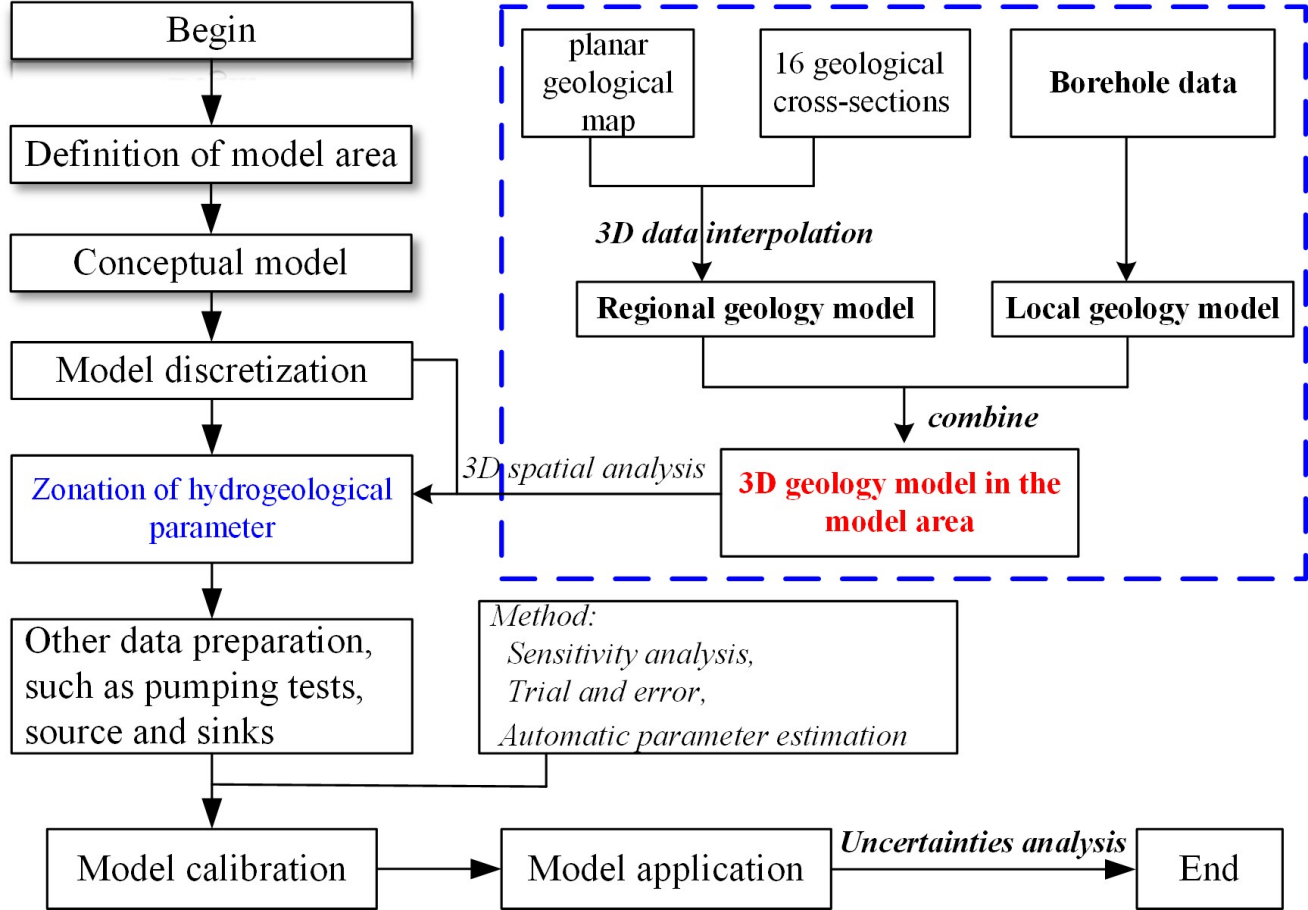
Flowchart of model development. Image created by the authors in Visio 2016; no copyrighted material was used.

The flowchart is shown in Fig 2. Firstly, the model area is defined and conceptual model is established. Secondly, model area is discretized using FEFLOW [26] software into grid systems, and a layered groundwater flow model is used. Thirdly, all data, including borehole logging, pumping tests data, local geology model and hydrogeological reports, are prepared, and then a 3D geology model will be developed regionally. Next, other model data is collected and used to carry out model calibration by pumping tests in the Musonoi area. Finally, the model is used to predict the dewatering flux for the underground Musonoi mine.

### Hydrostratigraphy and aquifer parameters

**Fig 3:**
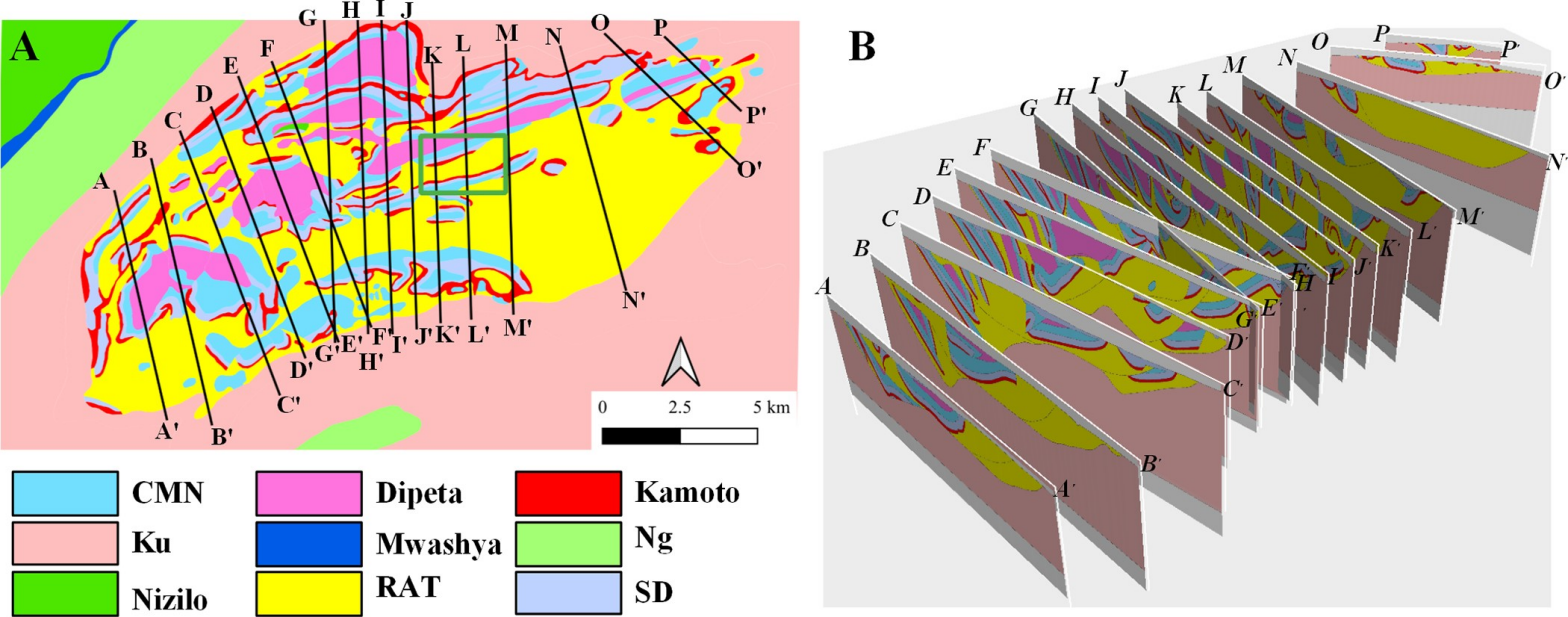
Lithological changes in the plain (A) and cross-sections (B) in the Kolwezi nappe area. Image created by the authors in QGIS 3.10, Datamine Studio RM 1.2.45.0 and Visio 2016; no copyrighted material was used.

The geological conditions are very complex in the study area, especially the Kolwezi nappe structure. According to previous geological research reports [20], 16 geological profiles (Fig 3A and 3B) are determined to divide the nappe bodies. The formation is reversed due to the presence of the overturned syncline, and the thickness differs in different locations. The geological modeling software is used to display the plane and section geological maps in the three-dimensional spatial coordinate system for better presentation (Fig 3B). Then, a three-dimensional geological model can be established to determine the parameters of the formation after raster map digitalization. The hydrogeological parameters are closely related to the structure and the degree of fragmentation caused by the stratigraphic structure. According to pumping test data, the hydraulic conductivity of the medium corresponding to the layer of CMN and RSF is approximately 0.65 m/d where the breccia zones are developed. The average hydraulic conductivity of the medium corresponding to the MWashya and RGS formation is 0.12 m/d, and the average hydraulic conductivity of media corresponding to the Kando and Kundelungu formation is approximately 0.026 m/d.

### Interaction between the rivers and the aquifer

According to the field investigation, the flow rates at each gauging station are basically the same from the start and end station along the flow of the Musonoi River. Considering the possible existence of drainage from the KOV pit to the mainstream of the river, it can be speculated that the hydraulic exchange between the Musonoi River and groundwater is present but not very strong. The interaction between the Musonoi River and groundwater is calculated by the groundwater level, the vertical hydraulic conductivity of the semipermeable underlying layer of the river, and the river stage [27], which is represented as the third boundary type in FEFLOW software. Little information about the seasonal Dilala River and the Luilu River is collected, and it is assumed that groundwater gains the recharge from the rivers and the infiltration rate is estimated by 5 to 10% of the runoff in this study.

### Sources and sinks

At the upper boundary of the system, the aquifer mainly receives recharge from precipitation, rivers, and the wastewater from tailing reservoirs. Groundwater discharge items mainly include mining drainage, drainage to abandoned pits, drainage to the river, pit leakage and groundwater evaporation. Groundwater evaporation occurs in the shallow depth areas of the groundwater. Recharge to the aquifer is mainly from precipitation during the rainy season. The precipitation infiltration coefficient is closely related to rainfall, the lithological structure of the aeration zone and buried depth of the groundwater level. According to previous studies, the rainfall infiltration recharge coefficient is generally between 10% and 25% but is only 3% in the RAT rock zone, 75% in some open pits, and up to 25% in other sandstone, waste craters and tailing ponds. In the model, the infiltration recharge rate is calculated by multiplying the annual average precipitation infiltration by the infiltration coefficient. Precipitation observational data are monitored by DRC Huakan Geological Services Co., Ltd. Groundwater abstractions from different mines are important items of groundwater drainage and have significant influences on groundwater level. The average dewatering flux was approximately 5.52×10^4^ m^3^/d in the Dikuluwe pit in September of 2018, and 4.22×10^4^ m^3^/d in the Mashambo west pit in September of 2018. The dewatering at these open pits is represented as the time-variant head boundary in the FEFLOW software, and the head is set by the current depth to the surface in each mine area.

### Water levels and pumping tests

**Fig 4:**
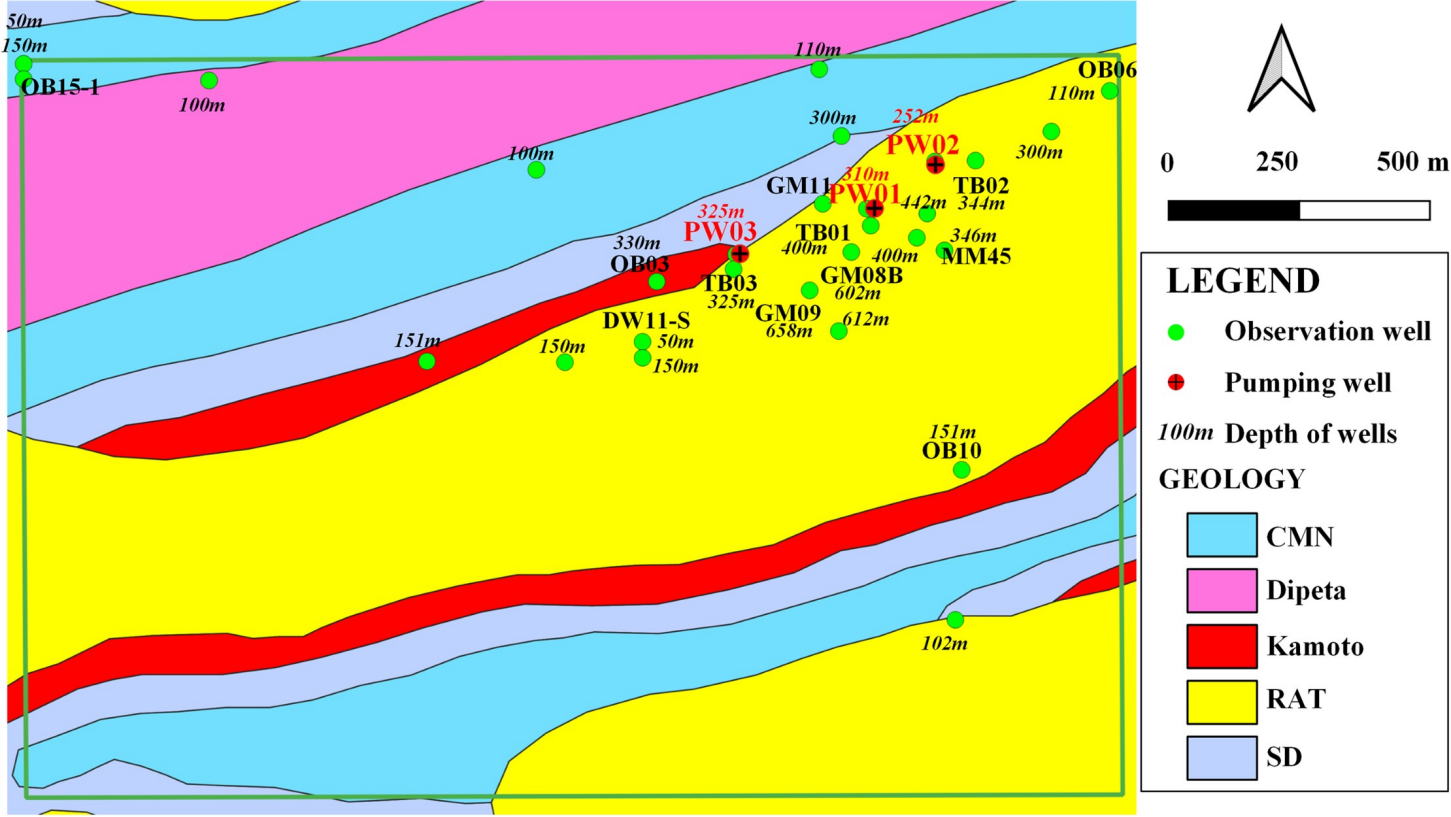
Distributions of observation wells in the area of interest, the number around the well means the depth of wells. Image created by the authors in QGIS 3.10 and Visio 2016; no copyrighted material was used.

Observation wells are distributed in the model area (Fig 1). To analyze the hydrogeological conditions of the Musonoi mine area and accurately estimate the dewatering of the Musonoi mine, a large-scale pumping test is carried out by DRC Huakan Geological Services Co., Ltd. The pumping tests begin at 8:00 a.m. on November 22, 2018, and stop at 8:00 a.m. on December 18, 2018. The restored groundwater level is observed from 8:00 p.m. of December 23, 2018. There are 3 pumping wells, PW01, PW02 and PW03, and the productions of each pumping well are 1232.4, 3532.32 and 2790.64 m^3^/d, respectively (Fig 4). There are 28 observation wells (including 3 pumping wells) in the area of interest. On December 18, 2018, the maximum drawdown among the wells is observed, approximately 61 m in the PW01 well, 58 m in the PW03 well and 45 m in the PW02 well. The drilling depths of each well are marked in Fig 4, and all wells are multilayered.

### Model domain, mesh and model layers

The model domain has an area of approximately 623.63 km^2^. The study area is divided into 12,740 nodes and 24,988 elements, with a total of 18 model layers (the total number of nodes is 242,060 and the number of elements is 449,784). In the study area, the region of the Kolwezi nappe is refined, and the Musonoi mine area is refined again. Observation wells and pumping wells are considered as fixed points, and the mesh is divided along the shape of the river network. The dry season in the study area is from May to September, and the time period for model calibration is set from September to December. The initial distribution of water level is thus calculated based on groundwater flow simulation under the steady state and then adjusts after the input of model data [27]. The model calibration is carried out based on the estimated hydrogeological parameters, the trial-and-error method and FePEST tool. The multilayered wells were represented as the multilayered well module in FEFLOW 7. The model adopts automatic time-step control via the forward Adams-Bashforth/backward trapezoid (AB/TR) time integration scheme.

### 3D geology model

**Fig 5:**
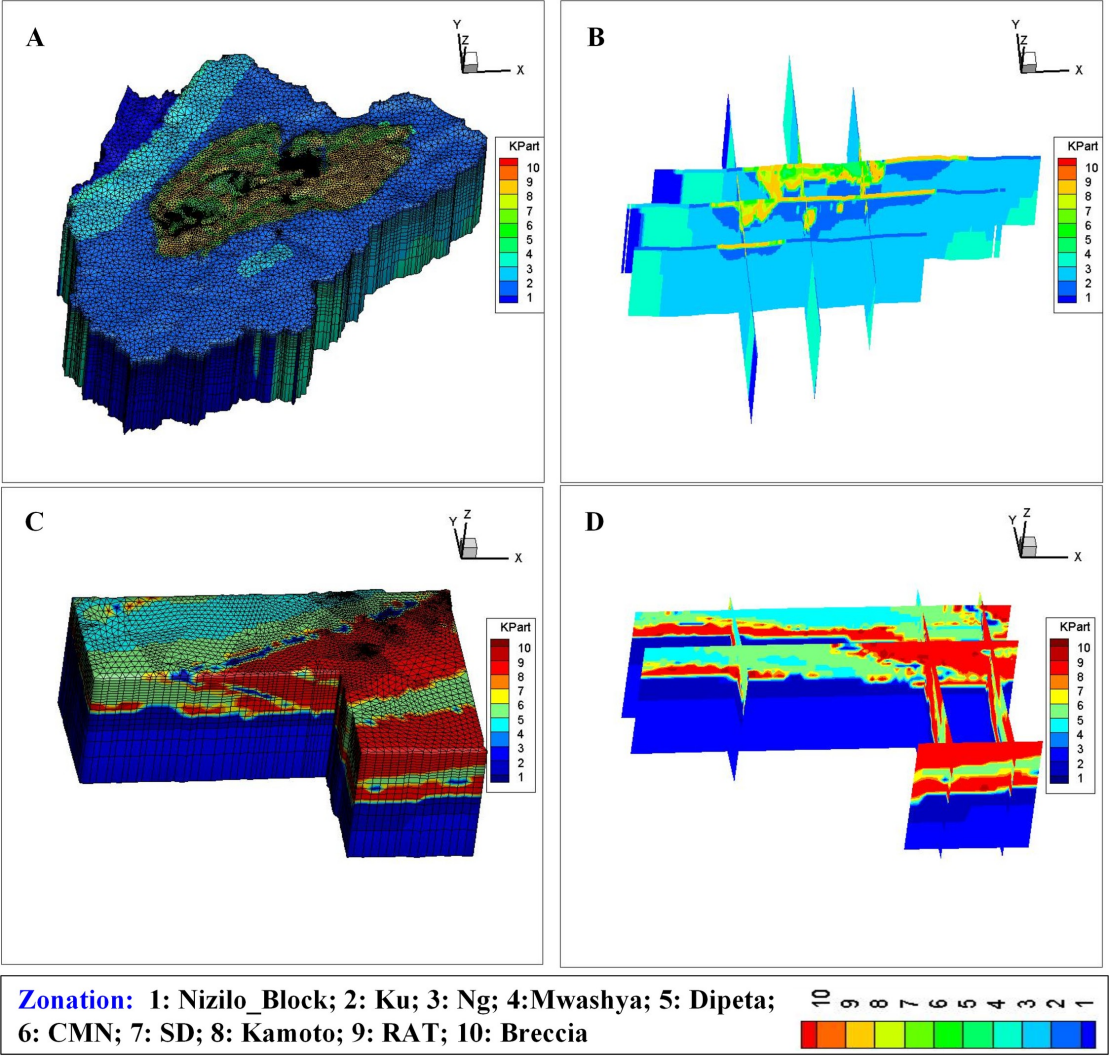
Demonstration of geology model in the model area and the Musonoi mine area, (A), (B), (C) and (D) means 3D mesh in the study area, cross-sections in the study area, 3D mesh in the area of interest and cross-sections in the area of interest, respectively. Image created by the authors in Tecplot 360 Ex 2014R1 and Visio 2016; no copyrighted material was used.

The ground elevation data used in this model are obtained by highly resolution elevation data from BIGEMAP software (http://www.bigemap.com/) with a horizontal resolution of 7 meters, which are publicly available. For the regional geology model, the depth range is taken from the ground surface down to 2000 m. The method is established based on grid systems as follows:

1. Establish a regional geological model in the study area. First, the plane geological map and 16 cross-sections are digitized. Then, the geological body is generated by using 3D data integration and Datamine RM software from all geological maps. The horizon clips of profiles at the 18 model layer depths are derived.
2. Develop a local geological model in the area of interest. The area of interest is approximately 6 km^2^ where the borehole logging data is used. The refined 3D geological model in the area of interest is produced using borehole data; the breccia zone is separately described in the geology model.
3. Couple the regional and local geology models and generate the zonation of hydrogeological parameters. The regional and local 3D geologies are coupled by the same grid system, and the partition of lithology in the study area is then formulated. Fig 5 shows the detailed zonation of hydrogeological parameters at the regional (Fig 5A and 5B) and local (Fig 5C and 5D) scales.

### Anisotropy and estimation of hydraulic parameters

High heterogeneity is present because of the overturned syncline in the area of interest. As indicated by the pumping tests, the maximum drawdown is approximately 61 m in the PW01 well, 18 m in the GM11 well and 18 m in the TB01 well; however, the distance between the pumping well of PW01 and the observation well is 130 m from the PW01 to the GM11 well and 44 m from the PW01 to the TB01 well, which suggests that anisotropy is still present in the study area and should be considered. According to the distribution of the formation, the angle between the direction of the anisotropy principal axis of hydraulic conductivity and the axis of the Cartesian coordinate system is generalized as the same value in the study area, and the angles of the principal axes are 60, 20 and 20 degrees from the *x*, *y* and *z* axes, respectively. The hydraulic conductivity of the long anisotropy principal axis in the plain is estimated by the pumping test data from engineering boreholes in the neighboring mine areas, and the value for each model layer is shown in Table 2. The hydraulic conductivity of the short anisotropy principal axis in the plain is set as the same as that in the long axis. The hydraulic conductivity of the vertical anisotropy principal axis is set at 1/10 of that in the long axis. Specific yield varies from 0.01 to 0.05 and is set to 0.02, 0.02, 0.04, and 0.05 for the CMN, SD, Kamoto, and Breccia zones, respectively. Storativity varied from 0.00001 to 0.0001 m^-1^ and is set to 0.0002, 0.0002, 0.0002, and 0.0001 m^-1^ for the CMN, SD, Kamoto, and Breccia zones (fractured zone), respectively.

**Table 2:** Estimation of hydraulic conductivity along the long anisotropy principal axis in the plain for each parameter zonation of each model layer in the study area (unit: m/d).

lithology	Nizilo_Block	Ku	Ng	Mwashya	Dipeta	CMN	SD	Kamoto	RAT	Breccia
Layer 1	0.0050	0.1307	0.0200	0.0200	0.0579	0.4803	0.3194	0.7500	0.0500	2.4000
Layer 2	0.0050	0.1307	0.0200	0.0200	0.0579	0.4803	0.3194	0.7500	0.0500	2.4000
Layer 3–4	0.0100	0.1307	0.0200	0.0200	0.1000	1.2000	0.4500	1.2000	0.2000	3.0000
Layer 5–8	0.0100	0.1307	0.0200	0.0200	0.0800	0.6500	0.3500	1.0500	0.1200	1.2000
Layer 9–11	0.0100	0.0984	0.0100	0.0100	0.0434	0.3600	0.2396	0.5625	0.0150	1.2000
Layer 12–13	0.0050	0.0660	0.0050	0.0050	0.0289	0.2396	0.1597	0.3750	0.0150	1.2000
Layer 14–15	0.0050	0.0660	0.0050	0.0050	0.0289	0.2396	0.1597	0.3750	0.0250	1.2000
Layer 16–18	0.0050	0.0050	0.0050	0.0050	0.0289	0.2396	0.1597	0.3750	0.0250	1.2000

## Results

### Sensitivity analysis of hydraulic conductivities and boundary conditions

Hydraulic conductivity (*K*) and recharge boundaries such as precipitation and surface water infiltration are critical factors for model calibration, and their uncertainties are required to assess. In this study, local sensitivity is calculated based on the single-factor variation method. The main factors include hydraulic conductivities in the Ku zone, the RAT zone, the Kamoto zone, the SD zone, the CMN zone and the Dipeta zone, and infiltration from rivers and reservoirs. The base scenario (*S*0) is set as initial estimated parameters. Scenarios are set by a factor with 25%, 75%, 125% and 150% of the initial parameters (shown in Table 3). The simulation period is set from 8:00 am on September 10, 2018, to 8:00 am on December 18, 2018. The sensitivity index (Eq (1)) is defined as the root mean square error of the simulated water level difference from between the scenario and the base scenario.

$$SA_{si}=\sqrt{\frac{1}{N}\sum_{j=1}^{N}{\left(h_{si}(j)-h_{s0}(j)\right)}^{2}}$$ (1)

**Table 3:** Scenario set of main factors for sensitivity analysis.

Factor	Ne (Abbreviation)	Scenarios			
		S1 (25%)	S2 (75%)	S3 (125%)	S4 (150%)
Hydraulic conductivity (*K*)	Ku (Ku)	Ku1	Ku2	Ku3	Ku4
	RAT (Ra)	Ra1	Ra2	Ra3	Ra4
	Kamoto (Ka)	Ka1	Ka2	Ka3	Ka4
	SD (Sd)	Sd1	Sd2	Sd3	Sd4
	CMN (Cm)	Cm1	Cm2	Cm3	Cm4
	Dipeta (Di)	Di1	Di2	Di3	Di4
Boundary Conditions	River and reservoir infiltration (Rech)	Rech1	Rech2	Rech3	Rech4

where *SA*_*si*_ is the sensitivity index for the scenario *Si* (*i =* 1,2,3,4), m; *N* is the total number of simulated values for 28 wells; *h*_*si*_ and *h*_*s0*_ are simulated water level from the scenario *Si* and the base scenario *S0*, respectively.

**Fig 6:**
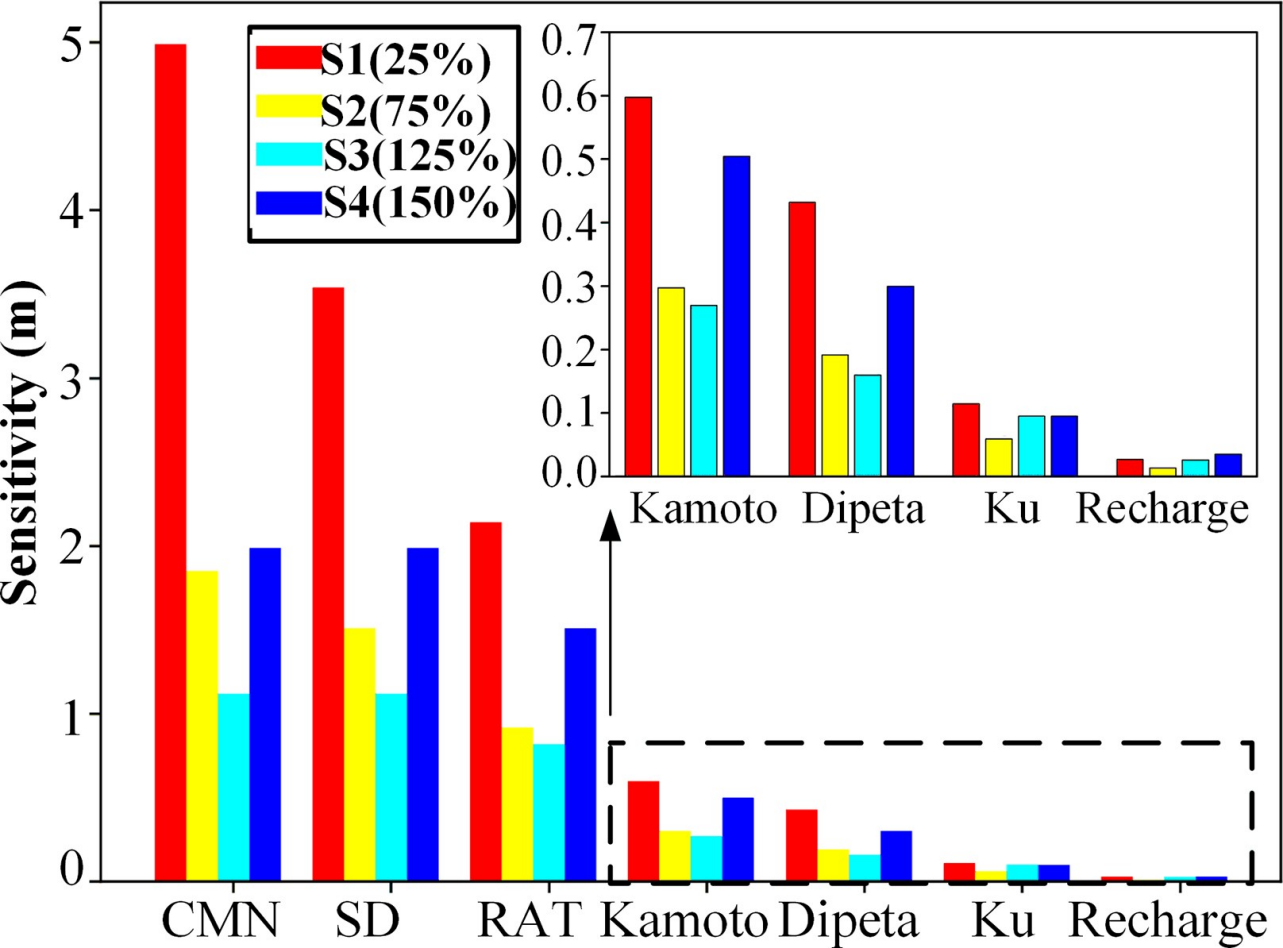
Local sensitivity of different geology zones and recharge conditions on water level changes. Image created by the authors in Python and Visio 2016; no copyrighted material was used.

Sensitivities of hydraulic conductivities for six zones, and surface water infiltration on groundwater level changes are shown in Fig 6. When hydraulic conductivities in the CMN, SD and RAT zones change, significant groundwater level changes are found, whereas hydraulic conductivities in the Kamoto, Dipeta, and Ku zones have smaller sensitivity. The scenario Cm1 has the biggest sensitivity (4.99 m). However, the sensitivity of river and reservoir infiltration is low (less than 0.10 m). So the hydraulic conductivities in the CMN, SD and RAT zones are the controlling factors which should be considered in the calibration period.

### Model calibration method

The calibration period is set from 8:00 am on September 10, 2018, to 8:00 am on December 18, 2018, for a total of 104 days. The pumping tests are carried out from 8:00 am on November 22, 2018, to 8:00 am on December 18, 2018. The main fitting targets are groundwater level data from 28 observation wells in the area of interest. The 28 observation wells mentioned above are selected as the fitting object to estimate the hydrogeological parameters. The objective function is set for this purpose as in Eq (2), and drawdown is used for metrics because the accurate head distribution in the Musonoi mine is not available. Under the constraints of the upper and lower limits of each parameter, the objective function *G* is the minimum. If the difference between simulated and observed water level during the calibration period is not sufficiently small, the values of some parameters are adjusted and the objective function is gradually minimized. First, the trial-and-error method was used to manually adjust the parameters. FePEST in FEFLOW was then used to estimate the parameters.

$$G(p_{1},\;p_{2},\cdots ,p_{n})=\sum_{i=1}^{N_{g}}\sum_{j=N_{tc}}\omega_{h}\left(i,\;j\right)•{\left[S\left(i,\;j\right)-S_{g}\left(i,\;j\right)\right]}^{2}$$ (2)

where *p*_1_, *p*_2_, …, *p*_n_ are the hydrogeological parameters to be estimated; *N*_g_ and *N*_tc_ are the number of observation wells and the time with maximum drawdown, respectively; *ω*_*h*_ is the weight of the groundwater level, here is set to 1 for 28 observation wells in the area of interest; *S*(*i*,*j*) is the simulated drawdown at the ^*j*^th time steps for observation well *i* (m), and *S*_*g*_(*i*,*j*) is the observed drawdown at the ^*j*^th time steps for observation well *i* (m).

### Comparison of observed and simulated drawdown

**Fig 7:**
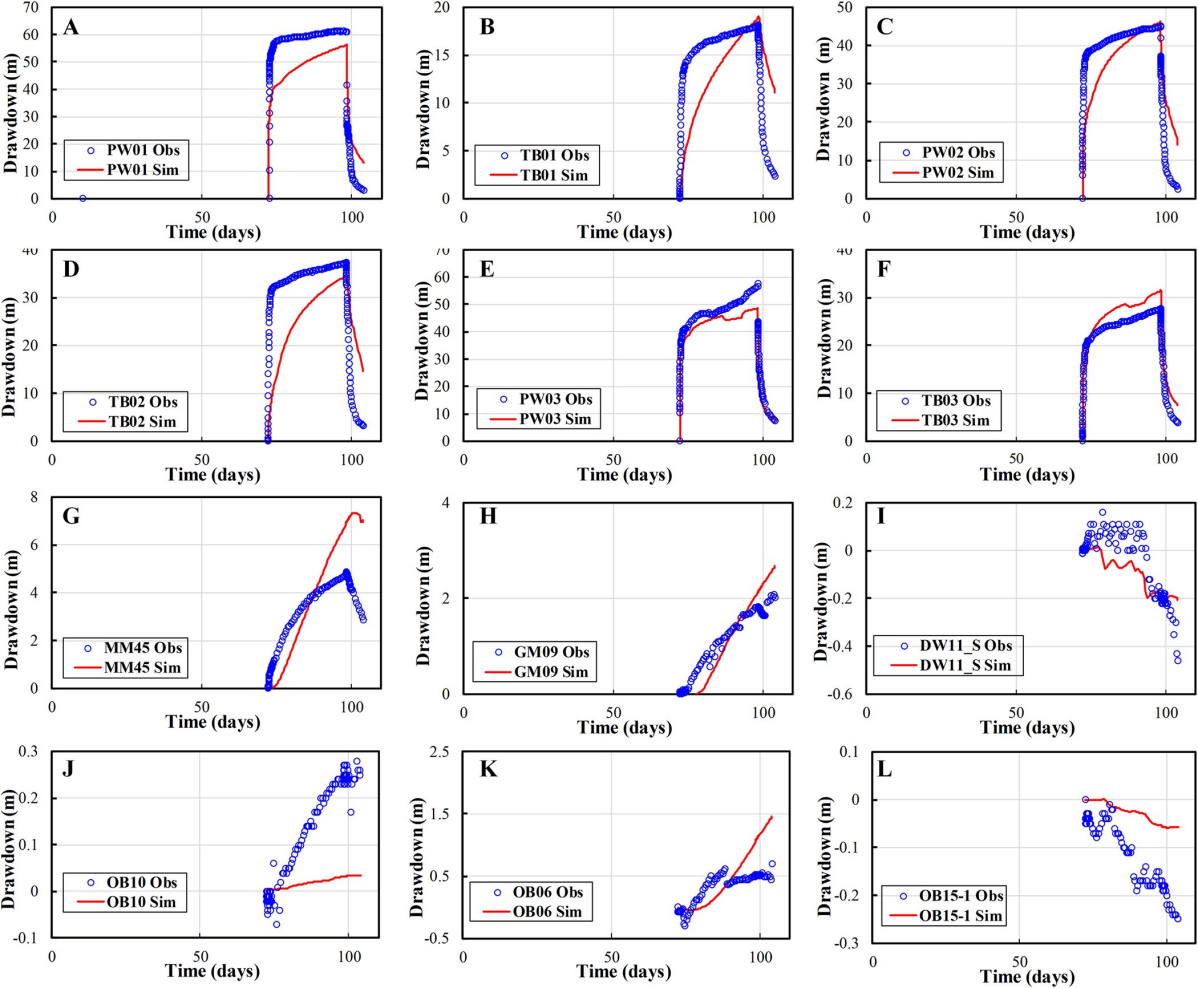
Comparison of simulated and observed drawdown in 12 observation wells. Image created by the authors in Excel 2016 and Visio 2016; no copyrighted material was used.

**Fig 8:**
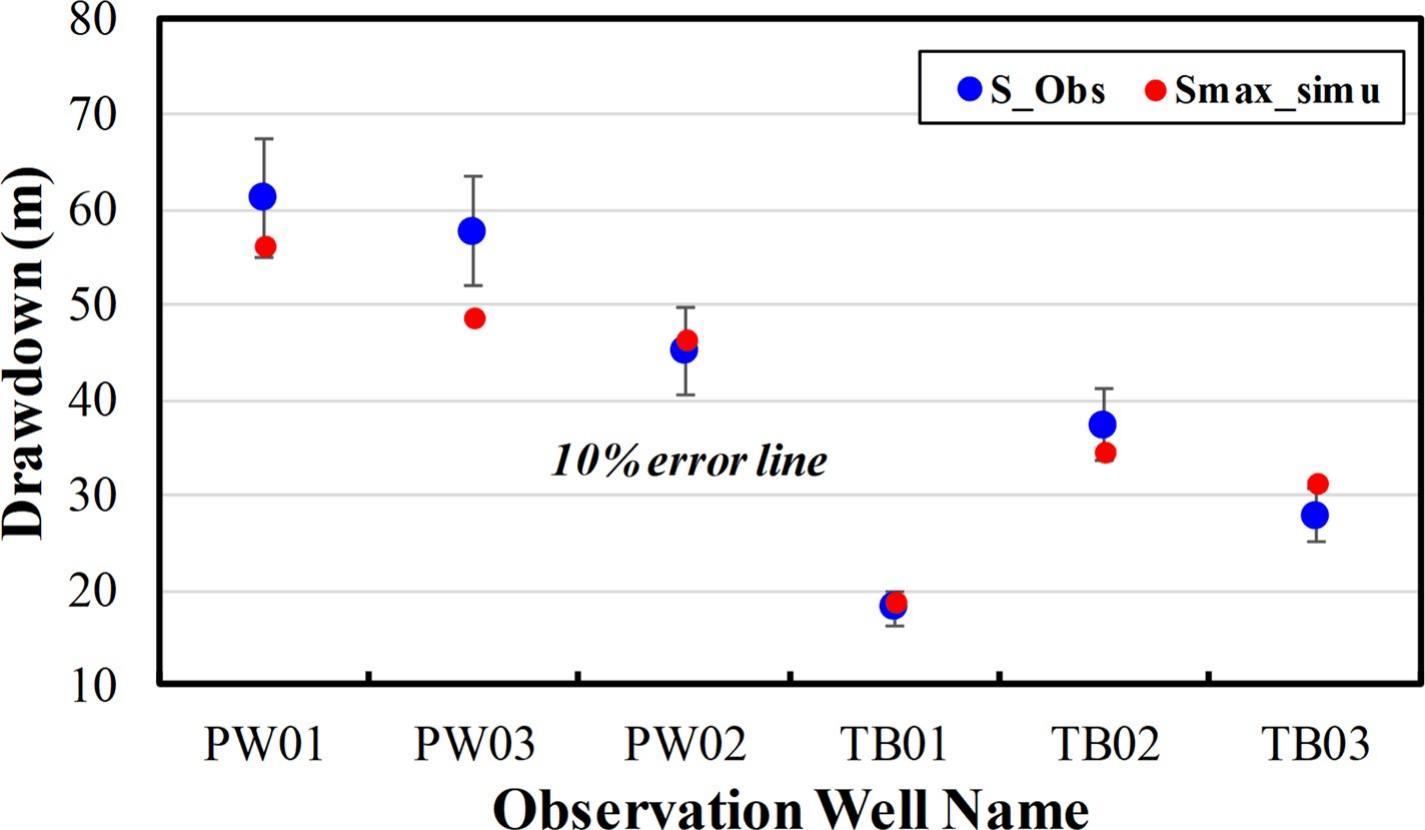
Comparison of the maximum drawdown during the pumping test for 6 wells. Image created by the authors in Excel 2016; no copyrighted material was used.

The simulated drawdowns are compared with the observed measurements, and the statistics are shown in Table 4. In the area of interest, approximately 41% of the absolute differences between the observed and simulated drawdown are within 0.5 m, approximately 56% of the absolute differences are less than 1.0 m, and approximately 22% of the absolute differences are bigger than 5.0 m. A total of 12 observed wells are selected to demonstrate the comparison of drawdown between the simulated and observed measurements over the calibration period (shown in Fig 7). During the pumping test period, the maximum drawdowns are located around wells PW01, PW02, PW03, TB01, TB02 and TB03. Fig 8 shows the comparison between the observed and simulated maximum drawdowns among six wells (PW01, PW02, PW03, TB01, TB02 and TB03) and the relative errors. The relative error of simulated maximum drawdowns is within 10% for wells PW01, PW02, TB01 and TB02 and approximately 15% for wells PW03 and TB03.

**Table 4:** Statistics of the absolute differences between observed and simulated drawdown among 28 wells.

Absolute difference between groundwater levels ΔS(m) in the Musonoi mine	ΔS≤0.5	0.5<ΔS≤1.0	1.0<ΔS≤5.0	ΔS>5.0	total
**Frequency**	**1655**	**624**	**879**	**902**	**4060**
**Percentage (%)**	**40.76**	**15.37**	**21.65**	**22.22**	**100**

### Calibrated hydraulic parameters

The hydraulic conductivity along the long anisotropy principal axis in the plain for each parameter zonation of each model layer in the study area after the calibration is shown in Table 5. The hydraulic conductivity along the long anisotropy principal axis is from approximately 0.05 to 0.43 m/d for the breccia zone, which is lower than the estimated value. Additionally, hydraulic conductivity from layers 5 to 8 is sensitive to the objection function in Eq (2) and is thus refined after calibration. The hydraulic conductivity of the short anisotropy principal axis in the plain is approximately 90% of the value in the long axis. The hydraulic conductivity of the vertical anisotropy principal axis is set at 1/15 of that in the long axis.

**Table 5:** Calibrated hydraulic conductivity along the long anisotropy principal axis in the plain for each parameter zonation of each model layer in the study area (unit: m/d).

lithology	Nizilo_Block	Ku	Ng	Mwashya	Dipeta	CMN	SD	Kamoto	RAT	Breccia
Layer 1	0.0015	0.0172	0.0029	0.0021	0.0132	0.1112	0.0784	0.1078	0.0463	0.1012
Layer 2	0.0009	0.0122	0.0022	0.0016	0.0083	0.0554	0.0420	0.0557	0.0240	0.1284
Layer 3–4	0.0018	0.0127	0.0041	0.0030	0.0136	0.0788	0.0068	0.0169	0.0199	0.1836
Layer 5	0.0012	0.0167	0.0030	0.0023	0.0202	0.0866	0.0217	0.0182	0.0252	0.2823
Layer 6	0.0012	0.0182	0.0030	0.0023	0.0639	0.1232	0.0358	0.0272	0.0365	0.4288
Layer 7–8	0.0012	0.0162	0.0032	0.0023	0.0361	0.0503	0.0339	0.0226	0.0272	0.1849
Layer 9–11	0.0012	0.0120	0.0021	0.0013	0.0145	0.0371	0.0283	0.0298	0.0126	0.0630
Layer 12–13	0.0007	0.0104	0.0014	0.0007	0.0158	0.0214	0.0180	0.0198	0.0126	0.0654
Layer 14–15	0.0006	0.0081	0.0007	0.0006	0.0084	0.0113	0.0130	0.0129	0.0048	0.0463

## Discussion

### Water table change and distribution of maximum drawdown

Usually, the initial head of each node is interpolated from the observed heads in observation wells. There are 18 model layers in the study area, and it is thus hard to obtain the initial head for all model layers because the wells are not well distributed in each layer and over the study area. The initial head is simulated by the coupled head and parameter method [27]. The cone of depression of the water table is clearly simulated and seen in the model area. In the area of interest, groundwater flows from the eastern and southern boundaries. Because the distance between the area of interest and the adjacent mines is short, the groundwater flow pattern will probably be altered after the intense mining activities in the area of interest, and it is uncertain whether the groundwater divide is present between the two mines.

**Fig 9:**
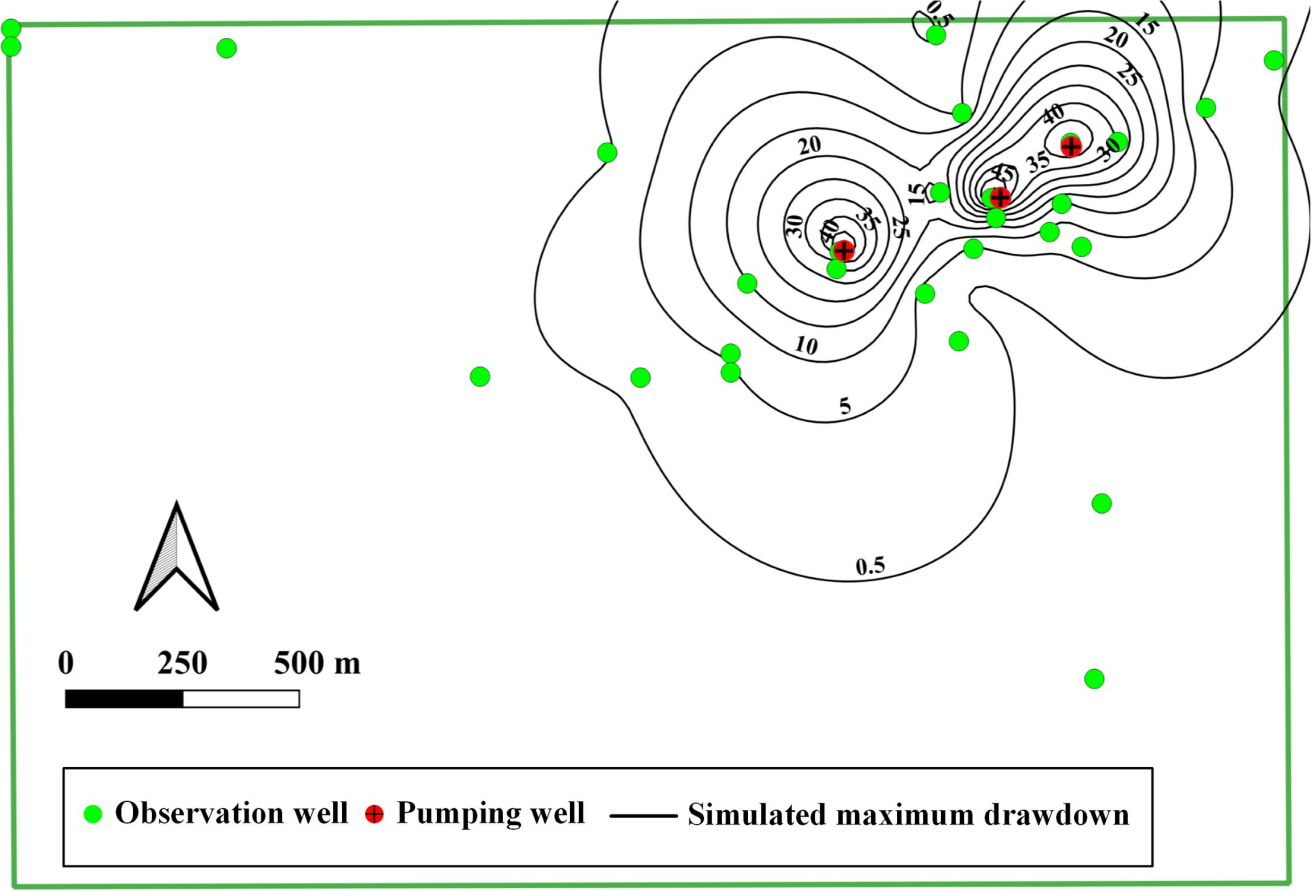
Contour map of maximum drawdown in the area of interest. Image created by the authors in QGIS 3.10 and Visio 2016; no copyrighted material was used.

The distribution of maximum drawdown during pumping tests can provide insightful understanding of dominant pathways for groundwater flow. The development of cone of depression in response to the pumping is mainly subjected to the area of the fault-controlled fracture zones (about the depth of about 150–350 m. Although the multilayered observation wells are not of the same depths as the boreholes, the maximum drawdowns of all wells are demonstrated in the plain (Fig 9). The distribution of maximum drawdown is highly un-uniform. The long axis of maximum drawdown is approximately 45° North of East, and the length of influence is approximately 1.50 km. The short axis of maximum drawdown is approximately 45° North of West, and the length of influence is approximately 1.0 km. Observation wells at the vertical depth of about 150–350 m have the biggest drawdown and then the wells in the shallow depth have the lowest drawdown.

**Fig 10:**
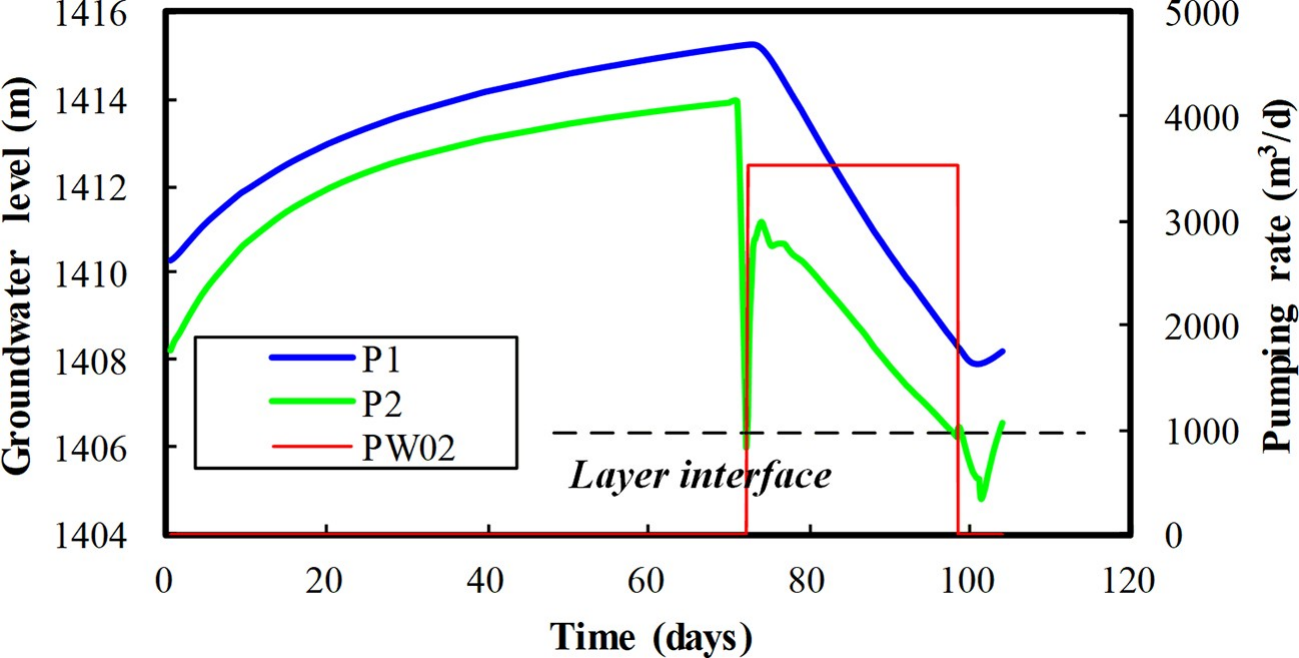
Observed drying and rewetting of the model layer. Image created by the authors in Excel 2016; no copyrighted material was used.

In the area of interest, the drawdown induced by dewatering is so large that drying of the aquifer usually happens. During the calibration, the phenomenon of drying and rewetting of the model layer (Layer 1) is observed (Fig 10). Under the influence of pumping well PW02, the change of groundwater level with time is normal in site P1 but abnormal in site P2 (here the locations of P1 and P2 are very close to PW02). The layer interface between model layer 1 and 2 is marked in Fig 10; two sudden water level jumps are present at the beginning and end of the pumping tests, which usually indicates convergence problems and low calculation efficiency.

### Dewatering flux prediction

**Fig 11:**
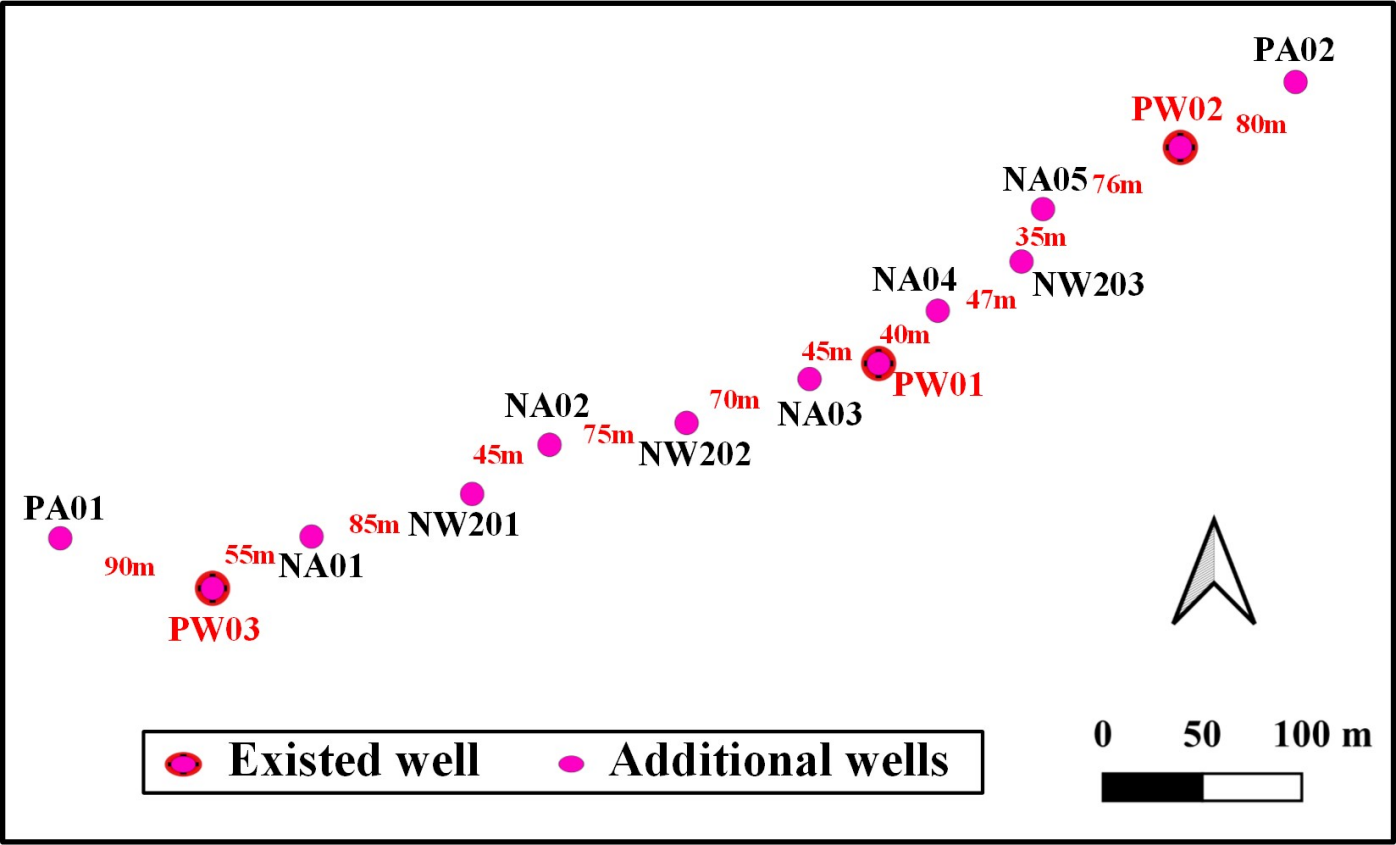
Layout of designed dewatering wells for 140 mL shaft. Image created by the authors in QGIS 3.10 and Visio 2016; no copyrighted material was used.

According to the draft mining plan, the first phase during the mine service period of the area of interest is to the depth of 140 mL and fully dewatered by pumping wells. Three pumping wells have been drilled during the period of pumping test, namely PW01, PW02 and PW03. The area of cone of depression under current pumping wells are relatively small, and additional wells should be added to extend the area. 140 mL dewatering plan will be achieved within 1 year, and 8 scenarios are thus considered (listed in Table 6). The depth of well PW01, PW02 and PW03 is 310.20 m, 251.51 m and 325.00 m, respectively. Well diameter of the three wells is varying with the depth from 0.780 m at the top to 0.378 m at the bottom. The diameter of well wall pipe at the bottom is 0.273 m for three pumping wells. In the model, the well radius is uniformly set as 0.189 m for three pumping wells. Under different scenarios for the 140mL shaft dewatering, the radius for all the newly added wells is set as 0.189 m, and the depth of these wells is set as 300 m. The scenarios S1 and S2 add the PB01, NW101, NW102 and PB02 wells, and the pumping rates are as shown in the table. The scenarios S3 and S4 add the PB01, NW201, NW202, NW203 and PB02 wells with pumping rates of 2000 m^3^/d, 2500 m^3^/d, 2500 m^3^/d and 2500 m^3^/d and 1500 m^3^/d, 1500 m^3^/d, 1500 m^3^/d and 2000 m^3^/d, respectively. The PB01, NA01, NA02, NA03, NA04, NA05 and PB02 wells with pumping rates of 2000 m^3^/d, 2500 m^3^/d, 2500 m^3^/d, 2500 m^3^/d, 2500 m^3^/d, 1500 m^3^/d, 1500 m^3^/d, 2500 m^3^/d, 1500 m^3^/d and 2000 m^3^/d, are added in the scenarios S5 and S6. The scenarios S7 and S8 add the PB01, NA01, NW201, NA02, NW202, NW203 and PB02 wells. The distribution of the dewatering wells in the six scenarios is shown in Fig 11.

**Table 6:** Dewatering scenarios set for 140 mL shaft.

Scenario	Well name / Pumping rate (m^3^/d)																										
	PB01			PW03				NW101					PW01			NW102						PW02			PB02		
S1	2000			2700				2500					1200			2500						3500			2000		
S2	2000			2700				2700					1200			3000						3500			2000		
	PB01					PW03			NW201					NW202			PW01		NW203				PW02			PB02	
S3	2000					2700			2500					2500			1200		2500				3500			2000	
S4	2000					2700			1500					1500			1200		1500				3500			2000	
	PB01	PW03			NA01		NW201		NA02		NW202			NA03		PW01		NA04		NW203			NA05		PW02		PB02
S5	2000	2700			2500		2500		2500		2500			1500		1200		1500		2500			1500		3500		2000
S6	2000	2700			1500		1500		1500		1500			1200		1200		1200		1500			1500		3500		2000
	PB01		PW03				NA01			NW201		NA02			NW202			PW01			NW203			PW02		PB02	
S7	2000		2700				2500			2500		2500			2500			1200			2500			3500		2000	
S8	2000		2700				1500			1500		1500			1500			1200			1500			3500		2000	

**Fig 12:**
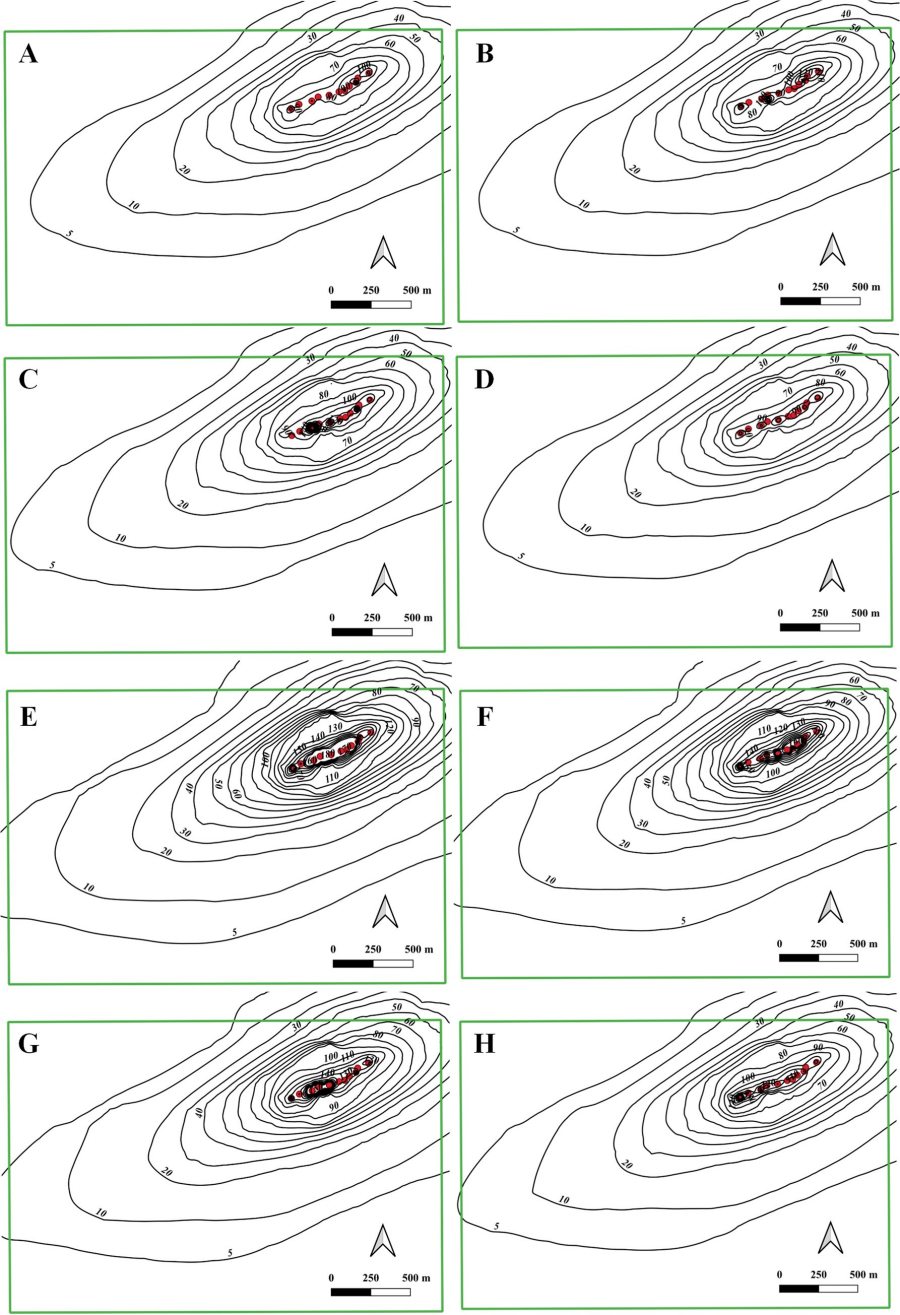
Contour map of drawdown after 1 year under 140 mL shaft for 8 scenarios, (A), (B), (C), (D), (E), (F), (G) and (H) represent the scenario 1, 2, 3, 4, 5, 6, 7 and 8. Image created by the authors in QGIS 3.10 and Visio 2016; no copyrighted material was used.

The simulated results under the eight scenarios are shown in Fig 12. The cone of depression with the drawdown of 140 m is required to cover the area of the mine area (as indicated by the red color in Fig 12). The areas of cone of depression under the eight scenarios are basically within the area of ore body, but the areas of the cone are different for scenarios. The contour map of drawdown pattern is an elliptical shape with a major axis from northeast to southwest. The scenario S5 has the largest area of the cone, while the scenario S1 has the smallest area. As can be seen from the figure, scenario S1, S2, S3, S4, S5, S6, S7 and S8 can form continuous cone area of drawdown with 80 m, 80 m, 90 m, 80 m, 150 m, 140 m, 120 m and 110 m, respectively. The total dewatering flux for scenario S1, S2, S3, S4, S5, S6, S7 and S8, is 16,400 m^3^/d, 17,100 m^3^/d, 18,900 m^3^/d, 15,900 m^3^/d, 28,400 m^3^/d, 22,800 m^3^/d, 23,900 m^3^/d and 18,900 m^3^/d, respectively. The scenario S5 have the highest dewatering flux, whereas the scenario S4 have the lowest flux. Although the area of cone of depression formed by the scenario S5 is large, the invest for drilling will be higher. So the scenario S7 is recommended when considering the area of dewatering and cost for the drilling and operation. It can be seen from the figure that the scenario S7 (Fig 12) can form a cone of depression with the drawdown of 120 m in the middle of 140 mL shaft after one year with maximum drawdown of 320 m, and the drawdown of 200 m at local regions.

Based on the simulated patterns of drawdown changes for the scenario S7, it can be found from the results that the cone of depression at the sixth model layer is the largest, and the maximum drawdown is nearly 320 m. The maximum drawdown decreases from the sixth model layer to the first model layer, and also from the sixth model layer to the eighth. From the existing pumping tests, the maximum production rate of PW03, PW01 and PW02 wells is 2700 m^3^/d, 1200 m^3^/d and 3500 m^3^/d, and the amount of production rate for additional wells in Table 6 can be reasonable. The total water volume of all wells in the middle section of 140 mL shaft is about 23,900 m^3^/d for the scenario S7. So the dewatering capacity with 23,900 m^3^/d will be required at least for the dewatering plan.

Pumping tests are carried out during the dry season, where the infiltration is about 2.19 mm/day. Different seasons such as normal season and wet season will also have an important influence on the dewatering. The model is also used to evaluate the influence of wet and normal season on the change of groundwater level. The precipitation is 1952 mm/year and 1200 mm/year for wet season and normal season, respectively, and then used as model input. More infiltration will increase groundwater level. For the scenario S7, relative to the dry season, the difference of drawdown is about 2–35 m under the wet season, and 1–30 m under the normal season in the area of interest. The dewatering activities will alter the water pressure conditions, and then engineering geologically safety such as collapse may occur. These geologically safety issue should be seriously paid attention during the underground mining. For the safety of dewatering, the change of groundwater levels should be monitored during the implement of dewatering project.

### Uncertainty of model results

Due to the high heterogeneity and anisotropy of geology formations, the simulated results have uncertainties. The main effects of the uncertainties are as follows:

1. Uncertainty of geological formations. Although this paper establishes the 3D geology model by coupling the regional 3D geological model based on geological cross-sections with a local refined geology model based on borehole data, local fractures may exist and the distributions of fractures remain still unknown due to limited field investigations.
2. Uncertainty of hydrogeological parameters. Pumping test data in the area of interest are used to calibrate the model. However, the three pumping wells are located at depths of 250–320 m below the ground surface, and observation wells are not well distributed in the area and in the vertical direction. The limited duration of a pumping test (less than 3 months in this study) is usually not sufficient to track the seasonality and changes in groundwater heads. From the calibration on pumping test data in the area of interest, obtained aquifer properties may not be representative over the whole extent of the model layers and model area.
3. Uncertainty of groundwater recharge. The model mainly uses the infiltration from precipitation in the dry season to estimate groundwater recharge, and the precipitation in wet years has different effects on the change of groundwater flow. No complete pumping test was conducted throughout the whole hydrological year, including the wet season, normal season and dry season. Therefore, the estimated dewatering may be underestimated.

## Conclusions

This study collects much data, such as precipitation, river runoff, groundwater level and the results of pumping tests, and constructs a three-dimensional groundwater flow model in an area of interest with a 3D geology model used to aid in the prediction of the dewatering of underground mines. Model calibration is conducted using pumping test data, and the characteristics of groundwater flow are discussed. The following conclusions are obtained.

1. Development of a 3D geology model for complicated formations can visualize the distribution of formations and facilitate the zonation of parameters in the groundwater models. The description of complex geological structure in the study area is challenging work. The geological information about sixteen hydrogeological cross-sections and the geology distribution in the plain are first digitized and then adopted to construct a regional geology model by using mining software. The local geology model in the mine area is refined based on borehole logging and fractures. Geology information from the two models is combined to formulate the 3D aquifer model for the study area. The description of the regional complex geological structure provides better conditions for improving the accuracy of the model.
2. A groundwater flow model was conceptualized, including the scope, the boundary conditions, the representation of multilayered wells, and sources and sinks. Although the area of interest is small, the model area is extended to the boundaries of the watershed to minimize the impact of the boundary conditions of the groundwater model on the accuracy due to the influence of intense mining activities. Vertically, the depth of 2000 m below the ground surface is regarded as the bottom impermeable layer of the model.
3. Model calibration is performed using pumping test data. From the fitting curves of groundwater levels, the model results are relatively good and could provide groundwater level changes due to dewatering in the area of interest.
4. Dewatering for 140 mL shaft is simulated by using the established groundwater flow model. Eight scenarios are set based on the existing three pumping wells (PW01, PW02 and PW03). In this study, it is recommended the scenario S7 with 7 pumping wells to dewater the aquifer, that is to say, adding four pumping wells with a depth of 300 m besides three wells. This scenario can best dewater the aquifer for the 140 mL shaft with the dewatering flux of about 23,900 m^3^/d. However, the engineering geologically safety, such as changes of water levels and engineering stability after dewatering, should be paid more attention during the operation of the mining activities.

This study provides a scientific method of model construction and initial predication of dewatering. However, model results may have some uncertainties. Due to the privacy of mines, detailed data, such as accurate dewatering data outside the area of interest, are difficult to achieve, and this may lower model accuracy to certain degree. During the current development of the Musonoi ramp, local fractures and veins data have been collecting from the front or wall and these parameters will be useful to contribute to the local geology model. Specify hydraulic conductivity of RSC layer whose generally have together with the CMN strongest transmissivity and water abundance below the weathering zones. The geology model may be improved at much refiner scales if ongoing investigations at small scales are finished.

## Supporting information

S1 File (XLS)

S2 File (DOCX)

## References

[1] Yihdego Y, Salem HS, Ayongaba B, Veljkovic Z. Mining sector challenges in developing countries, Tigray, Ethiopia and inspirational success stories from Australia. International Journal of Mining and Mineral Engineering 2018; 9(4): 321–367.

[2] Yihdego Y, Paffard A. Predicting open pit mine inflow and recovery depth in the Durvuljin soum, Zavkhan Province, Mongolia. Mine Water and the Environment 2017; 36: 114–123.

[3] Yihdego Y. Engineering and enviro-management value of radius of influence estimate from mining excavation. Journal of Applied Water Engineering and Research 2018; 6(4): 329–337.

[4] Bocheńska T, Fiszer J, Kalisz M. Prediction of groundwater inflow into copper mines of the Lubin Głogów Copper District. Environmental Geology 2000; 39: 587–594.

[5] Murdoch LC, Germanovich LN, Wang H, et al. Hydrogeology of the vicinity of Homestake mine, South Dakota, USA. Hydrogeology Journal 2012; 20: 27–43.

[6] Jiang SM, Kong XL, Ye H, et al. Groundwater dewatering optimization in the Shengli no.1 open-pit coalmine, Inner Mongolia, China. Environmental Earth Sciences 2013; 69: 187–196.

[7] Bahrami S, Ardejani FD, Aslani S, Baafi E. Numerical modelling of the groundwater inflow to an advancing open pit mine: Kolahdarvazeh pit, Central Iran. Environmental Monitoring and Assessment 2014; 186: 8573–8585. 10.1007/s10661-014-4025-x 25186026

[8] Yihdego Y, Danis C, Paffard A. 3-D numerical groundwater flow simulation for geological discontinuities in the Unkheltseg Basin, Mongolia. Environmental Earth Sciences 2015; 73 (8): 4119–4133.

[9] Hu LT, Jiao JJ. Modeling the influences of land reclamation on groundwater systems: a case study in Shekou peninsula, Shenzhen, China. Engineering Geology 2010; 114: 144–153.

[10] Schrader A, Winde F, Erasmus E. Using impacts of deep-level mining to research karst hydrology—a Darcy-based approach to predict the future of dried-up dolomitic springs in the Far West Rand goldfield (South Africa). Part 1: a conceptual model of recharge and inter-compartmental flow. Environmental Earth Sciences 2014; 72: 3549–3565.

[11] Bodeux S, Pujades E, Orban P, et al. Interactions between groundwater and the cavity of an old slate mine used as lower reservoir of an UPSH (Underground Pumped Storage Hydroelectricity): A modelling approach. Engineering Geology 2017; 217: 71–80.

[12] Mu WP, Wu Q, Xing Y, et al. Using numerical simulation for the prediction of mine dewatering from a karst water system underlying the coal seam in the Yuxian Basin, Northern China. Environmental Earth Sciences 2018; 77: 215.

[13] Pujades E, Suñé EV, Carrera J, et al. Dewatering of a deep excavation undertaken in a layered soil. Engineering Geology 2014; 178: 15–27.

[14] Benndorf J, Struzina M. Integrating geological uncertainty into planning and modelling for modern and efficient dewatering concepts in large surface mines. International Journal of Mining Reclamation and Environment 2014; 28: 231–241.

[15] Garzonio CA, Piccinini L, Gargini A. Groundwater modeling of fractured aquifers in mines: the case study of Gavorrano (Tuscany, Italy). Rock Mechanics and Rock Engineering 2014; 47: 905–921.

[16] Krčmář D, Sracek O. MODFLOW-USG: the new possibilities in mine hydrogeology modelling (or What is Not Written in the Manuals). Mine Water and the Environment 2014; 33: 376–383.

[17] DHI. Feflow finite element subsurface flow and transport simulation system -User's Manual. Recent release 7. 2. Technical report; 2018.

[18] Hassen I, Gibson H, Hamzaouiazaza F, et al. 3D geological modeling of the kasserine aquifer system, central tunisia: new insights into aquifer-geometry and interconnections for a better assessment of groundwater resources. Journal of Hydrology 2016; 539: 223–236.

[19] Mendelsohn. The geology of the North Rhodesian copperbelt. London: Macdonald 1961, pp351–405.

[20] François A. L'extremité occidentale del'arc cuprifère shabien Etude geologique Bureau d'études géologiques. Gécamines-Exploitation, Likasi, Zaïre Aulhenlie investment consulting (China) Lo. Ltd Translation in 2006 (in Chinese), 1973.

[21] François A. Carte géoogique de la region de Kolwezi-Kalukundi (Shaba, Republique de Zaire). Bulletin de la societe belge de Geologie 1980; 89: 141–143.

[22] Cailteux JLH, Muchez P, Cuyper JD, et al. Origin of the megabreccias in the Katanga Copperbelt (D.R.Congo). Journal of African Earth Sciences 2018; 140: 76–93.

[23] Nkulu CBL, Casas L, Haufroid V, et al. Sustainability of artisanal mining of cobalt in DR Congo. Nature Sustainability 2018; 1: 495–504. 10.1038/s41893-018-0139-4 30288453 PMC6166862

[24] Mukonki PM. Strategic mine planning: a SWOT analysis applied to KOV Open Pit Mine in the Democratic Republic of Congo. In World Academy of Science, Engineering and Technology International Journal of Geological and Environmental Engineering. 2017; Vol:11, No:11.

[25] Straskraba V, Placet J, Holubec M. Application of computer modeling for the design of open pit mine dewatering. IMWA Proceeding 1985; 579–597.

[26] Diersch HJG. FEFLOW—Finite element modeling of flow, mass and heat transport in porous and fractured media. Vol. XXXV Springer, Berlin Heidelberg, 2014, pp. 996. 10.1111/gwat.12285 25393965

[27] Chen CX, Pei SP, Jiao JJ. Land subsidence caused by groundwater exploitation in Suzhou City, China. Hydrogeology Journal 2003; 11: 275–287.

