# Supplementary material for: Estimating dewatering in an underground mine by using a 3D finite element model
Source: PLoS One. 2020 Oct 29;15(10):e0239682. doi: 10.1371/journal.pone.0239682 (PMC7595377; doi:10.1371/journal.pone.0239682)
Supplement: S2 File — (DOCX) [file pone.0239682.s002.docx]

Electronic supplementary material – Plos One

Estimating Dewatering in an Underground Mine by Using a 3D Finite Element Model

Litang Hu^1,2^*_,_ Menglin Zhang^1,2^_,_ Zhengqiu Yang^1,2^_,_ Yong Fan^3^*_,_ Jixiu Li^4^_,_ Hongliang Wang^5,6^_,_ Celestin Lubale^7^

^1^College of Water Sciences, Beijing Normal University, Beijing 100875, P.R.China

^2^Engineering Research Center of Groundwater Pollution Control and Remediation of Ministry of Education, Beijing Normal University, Beijing 100875, P.R.China

^3^ China ENFI Engineering Corporation, Beijing 100038, P.R.China

^4^Jinchuan Group Ltd, Beijing Road, Jinchuan District, Jinchang City, Gansu Province，P.R.China

^5^North China Engineering Investigation Institute Co., Ltd., Hebei Province 050020, P.R.China

^6^Technological Innovation Center for Mine Groundwater Safety of Hebei Province 050020, P.R.China

^7^Ruashi Mining SAS, Ruashi Mine Site, Tshimanga Street Number 18, Lubumbashi,Lualaba, the Democratic Republic of Congo

***** Correspondence: litanghu@bnu.edu.cn; fany@enfi.com.cn

**1. Pumping test dataset**

Filename: Pumping_test_dataset.xls

Including the location of 28 wells, pumping rates of three wells and changes of drawdown during the pumping tests.
